# Supplementary material for: Reversible inhibition of medial septum interneurons abolishes initial acquisition of new place-reward associations and reveals a progressive reduction in dependence on medial septal function
Source: bioRxiv. 2026 Oct 1:2026.09.25.754563. Preprint. [Version 1] doi: 10.64898/2026.09.25.754563 (PMC13648479; doi:10.64898/2026.09.25.754563)
Supplement: Supplementary file 1 [file NIHPP2026.09.25.754563v1-supplement-1.pdf]

bioRxiv preprint doi: https://doi.org/10.64898/2026.09.25.754563; this version posted October 1, 2026. The copyright holder for this preprint (which was not certified by peer review) is the author/funder, who has granted bioRxiv a license to display the preprint in perpetuity. It is made available under a CC-BY 4.0 International license.

Supplementary materials

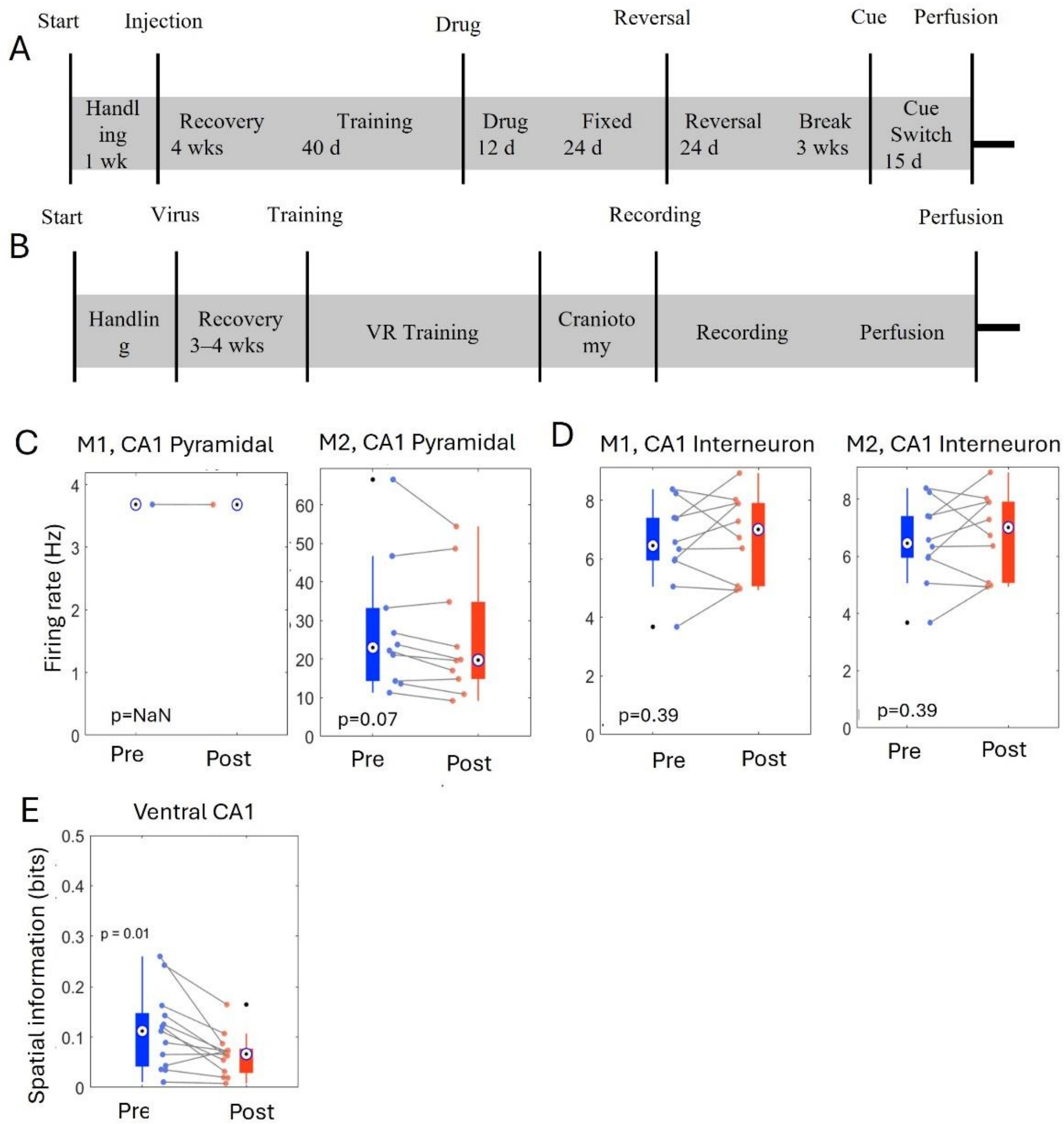

Supplementary Figure 1. Experimental timelines and effects of medial septal inhibition on ventral CA1 firing and spatial information.

(A) Behavioral timeline. Mice underwent 1 week of handling, 4 weeks of post-surgical recovery, 40 days of radial maze training, 12 days of alternating drug treatment, 24 days of fixed reward-arm training, 24 days of reversal learning, a 3-week break, and 15 days of post-delay cue-switch testing. (B) Electrophysiological timeline. Mice underwent handling, 3–4 weeks of post-surgical recovery, virtual reality (VR) training, craniotomy, recording sessions, and perfusion. (C) Firing rates of ventral CA1 pyramidal cells recorded before (Pre, blue) and after (Post, red) DCZ administration in two representative mice (Mouse1 and Mouse2). Each point represents one unit, with paired lines connecting pre- and post-DCZ values for the same cell. Boxplots show the distribution, median, and mean firing rate across units. (D) Firing rates of ventral CA1 interneurons recorded before (blue) and after DCZ administration (red) in the same two mice. (E) Spatial information of ventral CA1 pyramidal cells before (blue) and after DCZ administration (red).

bioRxiv preprint doi: https://doi.org/10.64898/2026.09.25.754563; this version posted October 1, 2026. The copyright holder for this preprint (which was not certified by peer review) is the author/funder, who has granted bioRxiv a license to display the preprint in perpetuity. It is made available under a CC-BY 4.0 International license.

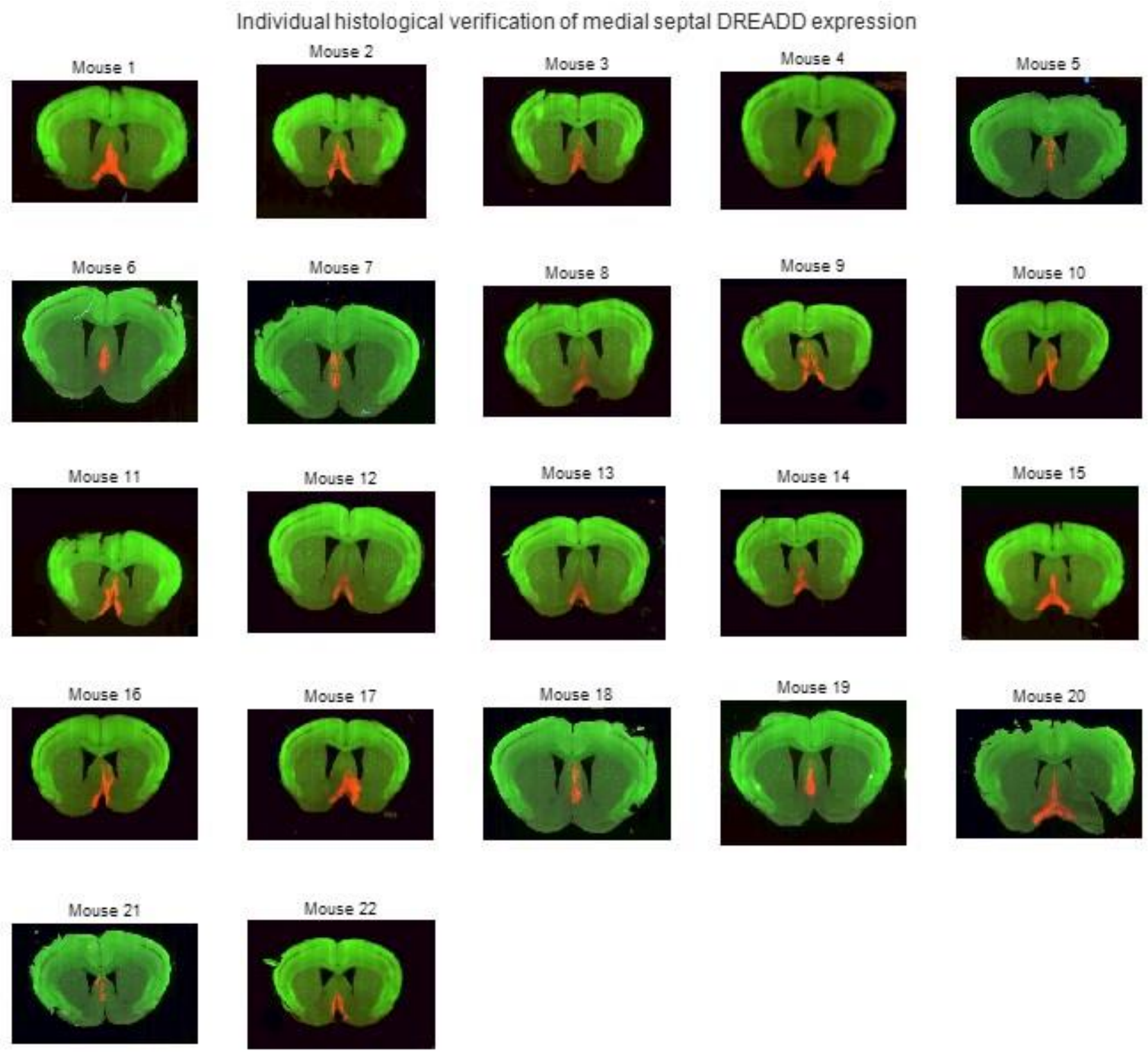

Supplementary Figure 2. Histological verification of medial septal DREADD expression in individual animals.

Representative coronal brain sections from individual mice included in the behavioral experiments, showing viral expression in the medial septal region. Green indicates tissue autofluorescence, and red indicates DREADD-associated viral expression. One representative section is shown for each animal (n=22).

bioRxiv preprint doi: https://doi.org/10.64898/2026.09.25.754563; this version posted October 1, 2026. The copyright holder for this preprint (which was not certified by peer review) is the author/funder, who has granted bioRxiv a license to display the preprint in perpetuity. It is made available under a CC-BY 4.0 International license.

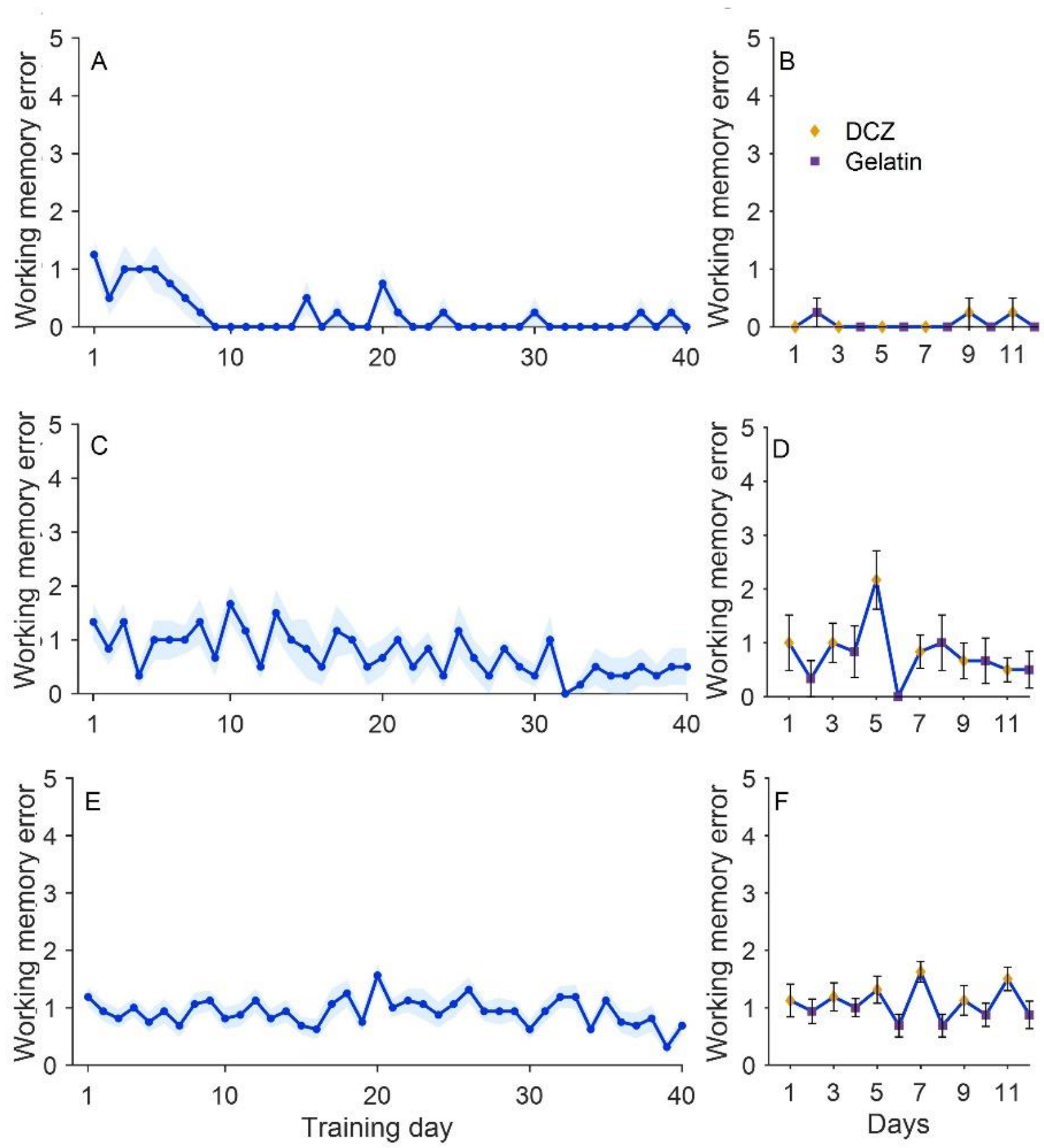

Supplementary Figure 3. Working memory errors across random reward-arm training.
(A) Mean working memory errors across 40 days of random reward-arm training in the control male cohort (n = 4). (B) Mean working memory errors across 12 alternating DCZ and gelatin sessions in the control male cohort (n = 4). (C) Mean working memory errors across 40 days of random reward-arm training in the first male cohort (n = 6). (D) Mean working memory errors across 12 alternating DCZ and gelatin sessions in the first male cohort (n = 6). (E) Mean working memory errors across 40 days of random reward-arm training in the second male cohort (n = 16). (F) Mean working memory errors across 12 alternating DCZ and gelatin sessions in the second male cohort (n = 16). In all figures, orange diamonds indicate DCZ sessions and purple squares indicate gelatin sessions. Error bars represent SEM.

bioRxiv preprint doi: https://doi.org/10.64898/2026.09.25.754563; this version posted October 1, 2026. The copyright holder for this preprint (which was not certified by peer review) is the author/funder, who has granted bioRxiv a license to display the preprint in perpetuity. It is made available under a CC-BY 4.0 International license.

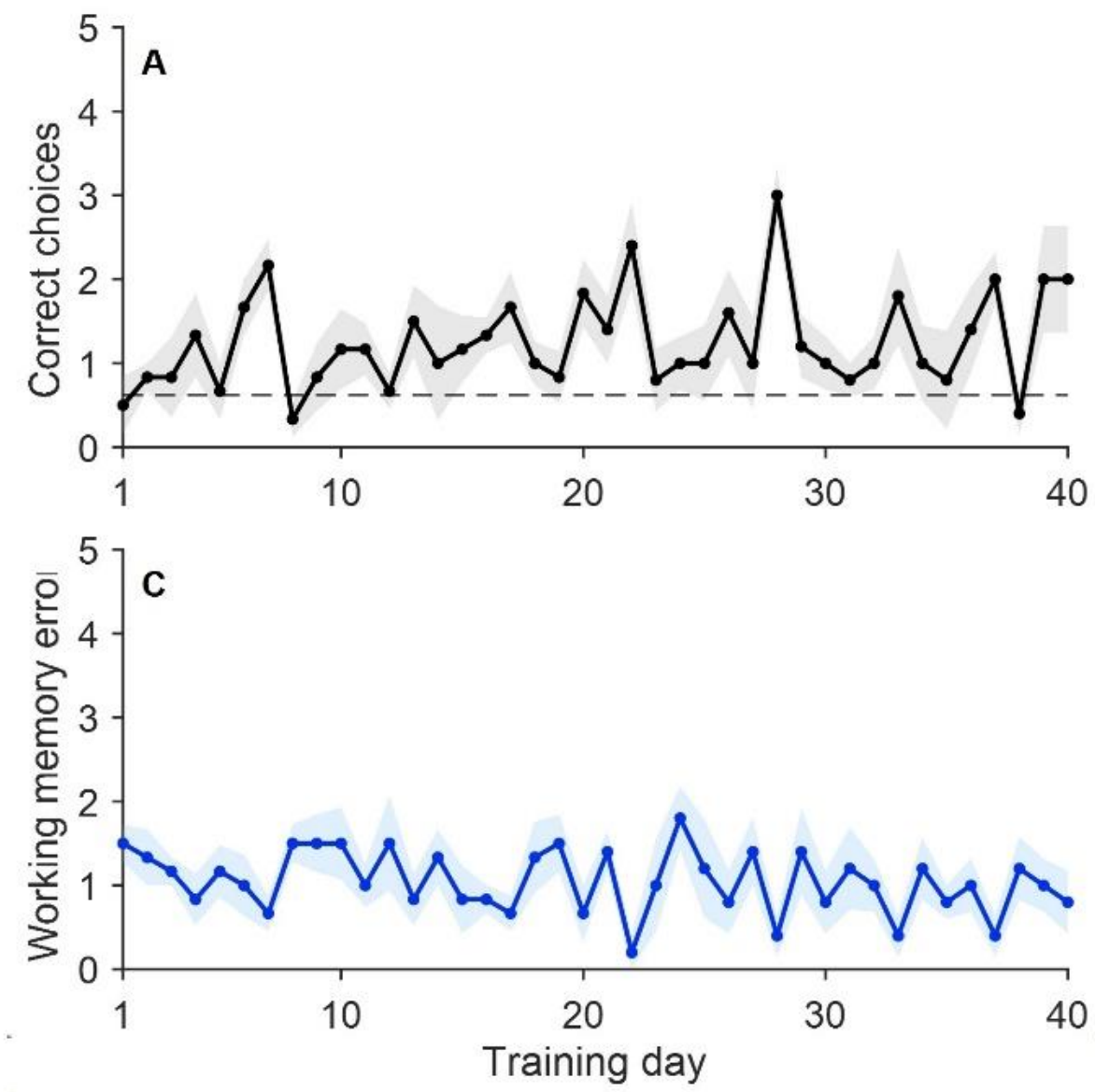

Supplementary Figure 4. Random reward-arm performance.

(A) Mean correct choices across 40 days of random reward-arm training in female mice (n = 6). (B) Mean correct choices across 12 alternating DCZ and gelatin sessions in female mice (n = 6). Orange diamonds indicate DCZ sessions and purple squares indicate gelatin sessions. Error bars represent SEM. The dashed horizontal line indicates chance performance (0.625). (C) Mean working memory errors across 40 days of random reward-arm training in female mice (n = 6). (D) Mean working memory errors across 12 alternating DCZ and gelatin sessions in female mice (n = 6).

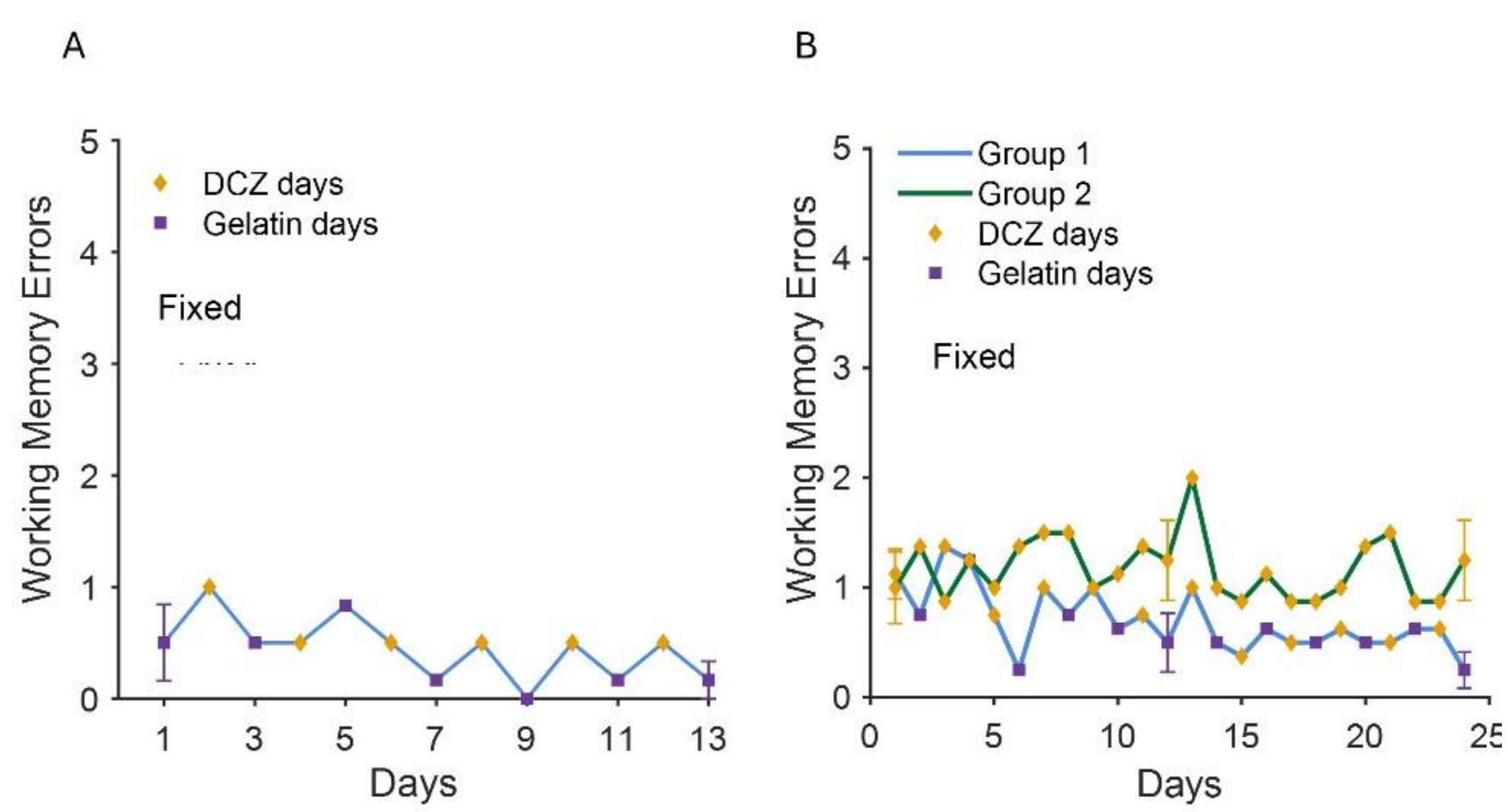

Supplementary Figure 5. Working memory errors during fixed reward-arm training.

(A) Mean working memory errors across 13 days of fixed reward-arm training in the first male cohort (n = 6). Orange diamonds indicate DCZ sessions and purple squares indicate gelatin sessions. Error bars represent SEM for treatment-matched days. (B) Mean working memory errors across 24 days of fixed reward-arm training in the second male cohort (n = 8 per group), shown separately for Group 1 and Group 2.

bioRxiv preprint doi: https://doi.org/10.64898/2026.09.25.754563; this version posted October 1, 2026. The copyright holder for this preprint (which was not certified by peer review) is the author/funder, who has granted bioRxiv a license to display the preprint in perpetuity. It is made available under a CC-BY 4.0 International license.

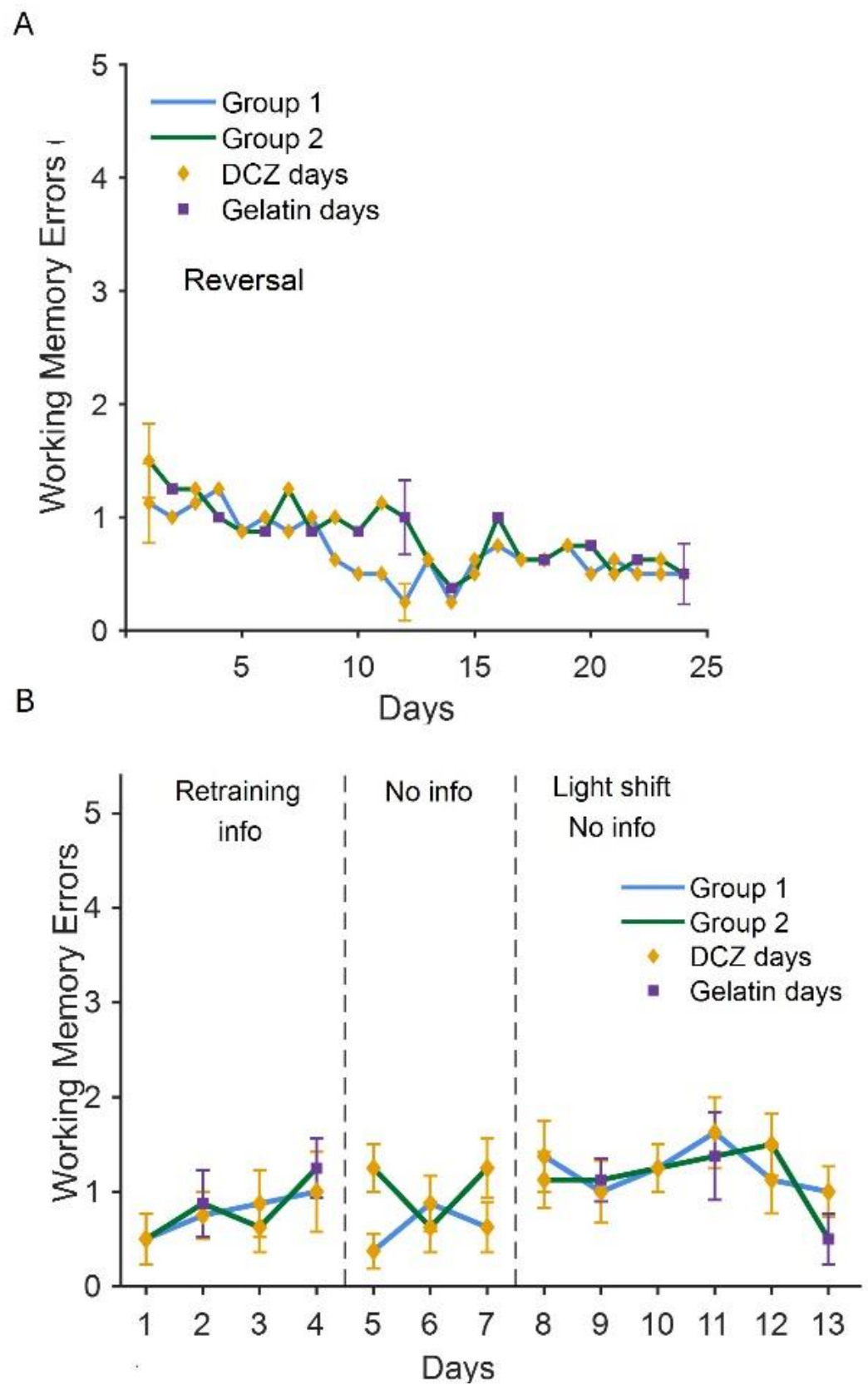

Supplementary Figure 6. Working memory errors during reversal learning and post-delay probe testing.
(A) Mean working memory errors across 24 days of reversal learning in the fixed reward-arm task (n = 8 per group). Group 1 and Group 2 are shown separately. Orange diamonds indicate DCZ sessions and purple squares indicate gelatin sessions. Error bars represent SEM for treatment-matched days.
(B) Mean working memory errors during post-delay retraining and probe testing following a 3-week break (n = 8 per group). Sessions are divided into retraining, no-information, and light-shift phases, indicated by dashed vertical lines. Group 1 and Group 2 are shown separately.

bioRxiv preprint doi: https://doi.org/10.64898/2026.09.25.754563; this version posted October 1, 2026. The copyright holder for this preprint (which was not certified by peer review) is the author/funder, who has granted bioRxiv a license to display the preprint in perpetuity. It is made available under a CC-BY 4.0 International license.

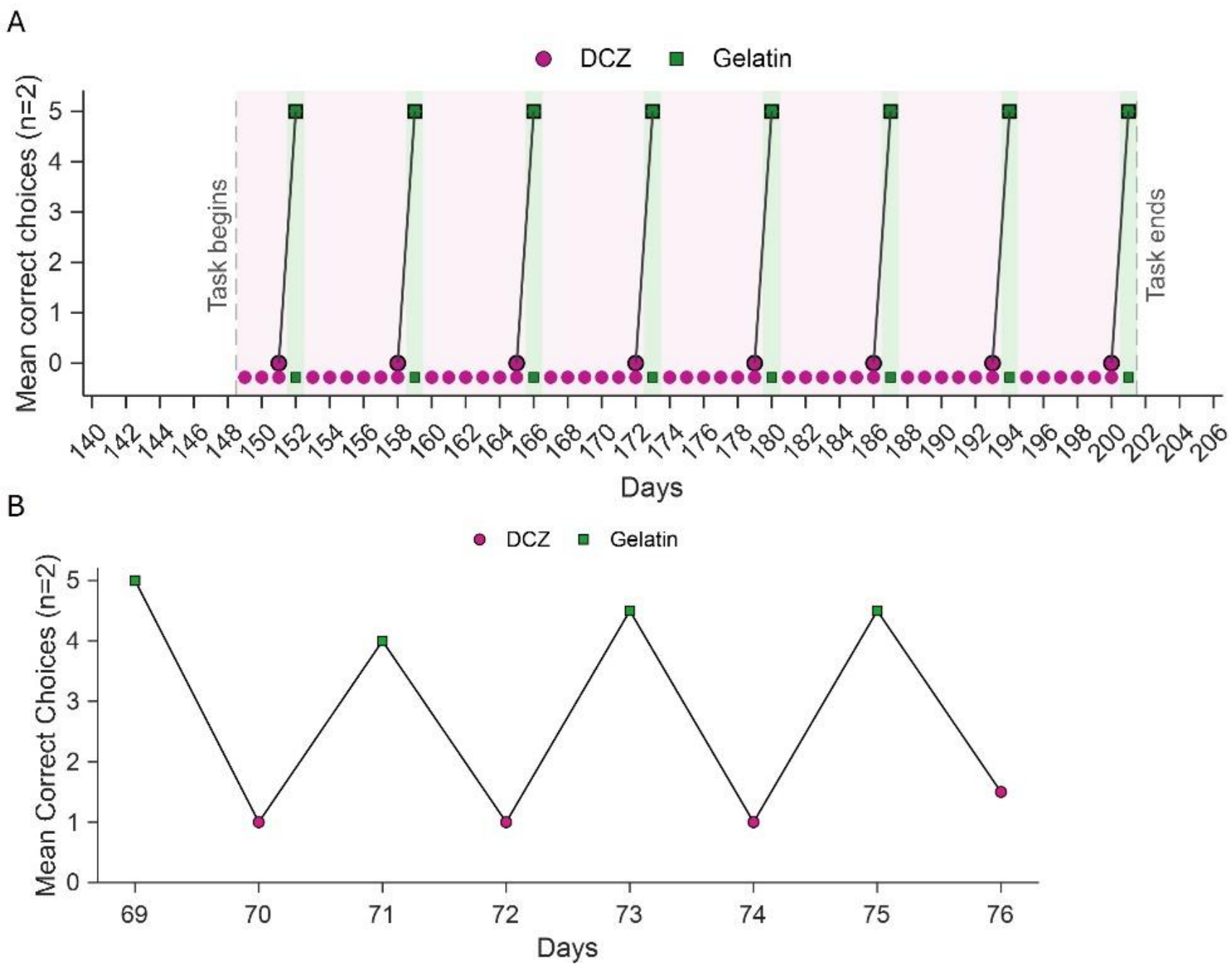

Supplementary Figure 7. Behavioral efficacy of DCZ was maintained across 200 days of repeated administration and remained evident when behavioral testing began 1 hour after administration.

(A) Two mice received 20 DCZ treatment sessions distributed across Days 1–147 (sessions not shown). Beginning on Day 148, DCZ was provided daily in the home cage, except on behavioral testing days, when mice received DCZ on one test day and gelatin on the other, according to the experimental schedule. (B) Mean number of correct choices across five trials during alternating DCZ (magenta circles) and gelatin (green squares) sessions. Behavioral testing began 1 hour after DCZ or gelatin administration. Lines connect consecutive testing sessions; *n* = 2 mice. Thus, the DCZ effect persisted long enough that waning drug effects cannot explain the gradual development of MS independence

bioRxiv preprint doi: https://doi.org/10.64898/2026.09.25.754563; this version posted October 1, 2026. The copyright holder for this preprint (which was not certified by peer review) is the author/funder, who has granted bioRxiv a license to display the preprint in perpetuity. It is made available under a CC-BY 4.0 International license.

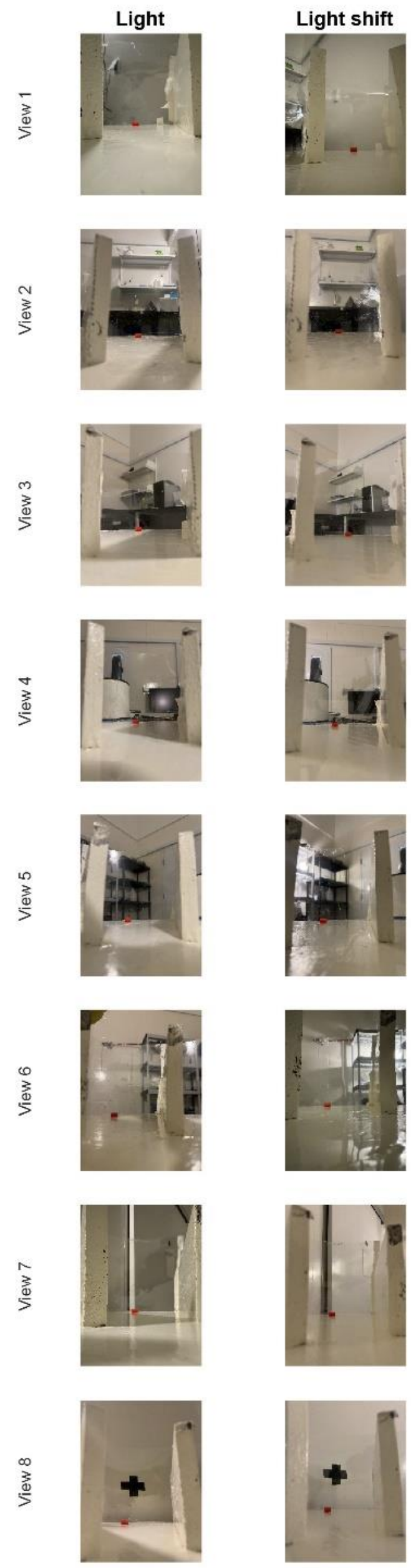

Supplementary Figure 8 . Visual environment before and after the 180° light shift.

Representative views of the experimental room from each of the eight arms of the radial-arm maze. Left, views under the original light configuration. Right, corresponding views after the light was shifted by 180°.
